# Quality of working life of professors in times of social distancing

**DOI:** 10.47626/1679-4435-2022-756

**Published:** 2022-03-30

**Authors:** Franciele Xavier da Silva, Jucileide Dias Oliveira, Leonardo Costa Pereira, Alexandra Daniela Marion-Martins

**Affiliations:** 1 Enfermagem, Centro Universitário Euroamericano (Unieuro), Brasília (DF), Brazil.; 2 Educação Física, Unieuro, Brasília (DF), Brazil.

**Keywords:** quality of life, faculty, social isolation, pandemics, COVID-19

## Abstract

**Introduction:**

Quality of working life consists in a dynamic management of physical, technological, and sociopsychological factors that affect culture and renovate the organizational climate, reflecting on workers’ wellbeing.

**Objectives:**

To identify changes in eating habits, physical activity, and hours of sleep, as well as their influence on the quality of working life of professors who shifted to remote working during the pandemic.

**Methods:**

This study has a quantitative, descriptive, observational design and was performed with faculty members of a private higher education institution in May 2020; 40 professors participated in our research. We used a virtual questionnaire containing Walton’s quality of working life scale (1973) and questions on lifestyle habits.

**Results:**

We observed that changes in lifestyle habits were correlated with lower or higher perceptions of satisfaction with the 4 evaluated criteria referring to quality of life: working conditions, use of capacities at work, social interaction at work, and the space work occupies in life.

**Conclusions:**

The mean quality of working life score of faculty members working remotely during lockdown demonstrated that, despite changes in work and lifestyle habits, professors still maintained a certain degree of satisfaction with the aspects of work evaluated in this study.

## Introduction

Quality of working life (QWL) consists in the dynamic management of physical, technological, and sociopsychological factors affecting organizational climate and promoting workers’ wellbeing. In addition to factors intrinsic to work, personal habits may interfere with an increase or reduction in worker satisfaction. Habits with a positive impact on QWL are related to adequate sleep, healthy eating, and regular physical activity.^1^ Therefore, when evaluating QWL in the workplace, it is necessary to observe the various aspects permeating workers’ lives. Changes at work such as routine, rhythm, pressure, shifts, hours, and challenging situations may affect QWL.^2^

The pandemic caused by the severe acute respiratory syndrome coronavirus 2 (SARS-CoV-2), which in Brazil started in February 2020, and social distancing altered the routine of a large part of the Brazilian population and changed learning methods. Part of the country’s higher education institutions (HEI) interrupted their activities, whereas others soon resumed their activities remotely.^3^ Learning activities thus switched from in-person to remote. According to the Brazilian Ministry of Education, distance learning “is the educational modality where the didactic and pedagogical mediation of teaching and learning processes occurs through the use of means of communication and information technologies, with students and teachers developing educational activities at different times and places”.^4^

The adaptation of learning to social distancing was performed in a diversified manner, in order to respond to the students’ demands and to the possibilities available to professors. Virtual learning was conducted through synchronous and/or asynchronous activities. Synchronous activities were performed with the presence of the professor and student at the same time, whereas asynchronous activities used recorded activities or lectures without meetings with the professor.^5^ Considering the concept coined by the Ministry of Education, the learning modality adopted during the pandemic differed from purely distance learning because the presence of a professor remained as an important link with the student. The possibility of recording lectures so that students could access them at different times was a strategy for minimizing losses for those who did not have internet access at the time of the class or who had household tasks that prevented them from watching synchronous classes.^6^

According to the 2019 census performed by the National Institute for Educational Studies and Research (INEP), there were 386 073 active faculty members at 2608 Brazilian HEI. For these professors, work done at home (also named remote working or telework) required a place in their residence for performing it, in addition to a digital tool for communicating with the student, and internet access.^5^ Some HEI provided professors with electronic learning platforms; however, other institutions transferred the responsibility of discovering communication applications (free or paid for by themselves) to the professors. Some of these professors did not have experience with digital communication technologies and had to quickly learn how to use them to continue teaching. These challenging situations may have required a higher emotional, physical, and financial dedication to work.^7,8^

Apart from remote working, the introduction of social distancing changed people’s routines. Lack of socialization due to lockdown, constant bad news on media outlets, and changes in hygiene protocols may have also had a psychological impact on professors and their work productivity.^8^ University professors are one of the most psychologically affected professions due to long weekly hours, work overload, unpreparedness to meet changes and demands of work, excessive bureaucracy, and precarious working conditions.^9^ The overload previously reported by studies may have been increased with the need to prepare lectures using virtual tools and to answer students’ demands outside of class hours. Precarization of the work environment may have occurred due to lack of technological support and material, lack of autonomy, and hierarchical pressure.^10^

Bearing in mind the importance of this significant working class, the impact of social distancing on education and on the workers involved, and a better understanding of changes in their lifestyle and work process, it is important to study the QWL of professionals working remotely during the pandemic. The hypothesis of this study is that during the pandemic, faculty members went through changes in lifestyle habits; these, when combined with changes in the remote work process, had an impact on job satisfaction. Considering that QWL encompasses various factors and that personal situations and perceptions interfere with job satisfaction, this study aimed to identify changes in eating habits, physical activity levels, and hours of sleep, as well as their influence on the QWL of professors who shifted to remote working during the pandemic.

## Methods

### Study design

This is a cross-sectional observational study with a quantitative inferential approach. The study was performed in a private HEI in the Brazilian Federal District in May 2020. All information in this study was presented according to the Strengthening the Reporting of Observational Studies in Epidemiology (STROBE) guidelines. Our methods were in accordance with the criteria for research with human subjects of the Ministry of Health, Directive 510/2016. The informed consent form (ICF), with clarifications about the research, was provided before presentation of the questionnaire, and acceptance (or not) was mandatory for continuing with questionnaire filling. This research was approved by the Research Ethics Committee, opinion No. 4.008.696.

### Context

The HEI was chosen for convenience in recruiting participants and due to its control of the transition from in-person to remote learning. At the time of the study, the HEI offered 28 undergraduate courses and 15 graduate courses throughout 3 campuses. It held a team of 248 professors (109 women and 139 men). Faculty members started remote activities in March 2020. The institution provided the Microsoft Teams platform for the classes to continue. The know-how for remote working was acquired through workshops and the exchange of information between faculty members. The study was performed after approximately 2 months of remote working.

### Participants and study size

All active professors working with remote classes for the HEI were eligible for participating in the study. We included those who filled in the form and agreed to participate by expressing acceptance in the ICF. No professors were excluded from our analysis. Convenience sampling totaled 40 faculty members from 17 courses and 3 different campuses.

### Variables

Variables were grouped into QWL, behavioral patterns before and during the pandemic, and socioeconomic characteristics. We used the following variables to characterize the sample: sex, age, area of expertise, weekly working hours, years of experience in teaching, activities performed at the HEI, and additional occupation. Variables related to behavioral patterns before and during the pandemic were the intake of fruits and vegetables, home-delivered meals, physical activity, and hours of sleep. Finally, variables related to QWL were working conditions (C1), use of capacities at work (C2), social interaction at work (C3), and the space work occupies in the professor’s life (C4).

### Measurements

We used the translated and validated Portuguese version of Walton’s instrument (1973) for evaluating QWL, obtaining a Cronbach’s alpha of 0.96.^11^ This model comprises 8 criteria. The instrument was adapted for this study, maintaining 4 criteria that assess work-related aspects which suffered changes when switching from in-person to remote work. We used a Likert scale from 1 to 5, where a score of 5 stood for “very satisfied” and a score of 1 meant “very unsatisfied.”

### Data sources

The data collection tool was constructed using the Google Forms platform, containing a semi-structured questionnaire tested in a pilot study and refined for better understanding by the participants. The first section presented clarifications about the research and the ICF, with the option of accepting or declining to participate in the study. The second section comprised questions characterizing the sample, its lifestyle habits, and QWL, totaling 36 questions. The questionnaire was sent to the email addresses of the institution’s professors, as well as in group chats of the WhatsApp text messaging app, in order to facilitate access. This procedure was repeated twice due to a low response rate.

### Statistical methods

We used the Statistical Package for the Social Sciences (SPSS), version 22.0, for data analysis. Data from categorical variables were presented as absolute and/or relative frequencies; data from numerical variables were presented as measures of central tendency (mean and median) and their respective measures of dispersion (standard deviation and interquartile range). For comparing different groups of professors stratified according to behavioral analyses (decreased, maintained, or increased behaviors), we used the Kruskal-Wallis test. In case a significant difference was detected, we used the Mann-Whitney test as a post-hoc analysis. Spearman’s correlation was used to identify possible correlations between domains of quality of life. This study adopted an alpha level of 5% for characterizing significance.

## Results

Professors who answered the questionnaire represented 16% of all faculty members at the studied HEI. Most participants were female, aged between 30 and 39 years, had 8 or more years of experience, and worked at the HEI for at least a year. Forty professors were part of the faculty of 17 courses. The mean weekly number of teaching hours was 19.45, the lowest workload was 5 hours, and the highest workload was 34 hours. Most professors (58%) had other activities at the HEI in addition to teaching hours, such as supervising undergraduate dissertations, outreach activities, research internships, laboratory, supervised internships, management, or taking part in the Structuring Faculty Center (Núcleo Docente Estruturante, NDE). In addition to professorship, 48% of them had other jobs.

Before the pandemic, most professors (70%) reported some kind of physical activity, all of them ate fruits and vegetables at least twice a week, half of them did not eat home-delivered meals, and approximately one-third slept for 5 hours a day or less. After 2 months of social distancing, 52.5% of the professors reported not doing any physical activity, part of them (12.5%) reported having stopped eating fruits and vegetables, 62.5% began consuming home-delivered meals, and most of them (80%) stated they slept for more than 6 hours a day.

**Figure 1:**
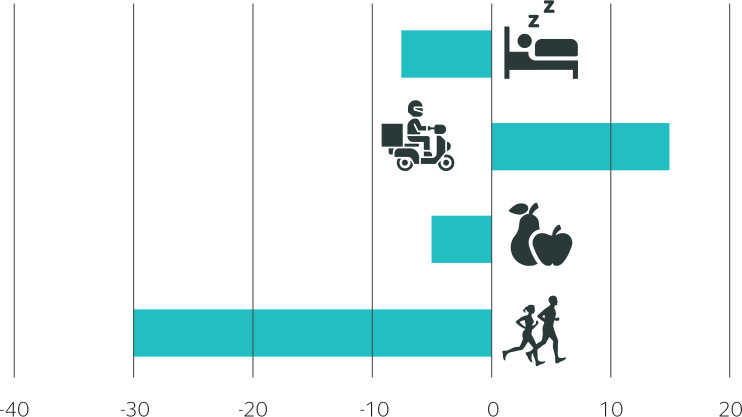
Alterations in physical activity, eating, and sleeping behaviors of professors who switched to remote teaching after the beginning of the COVID-19 pandemic, presented as relative frequencies. From the top: columns represent variations in hours of sleep, food delivery orders, fruit and vegetable intake, and physical activity.

When analyzing habit changes per individual, we noticed that in general, the amount of sleep decreased, food delivery orders increased, the fruit and vegetable intake decreased, and physical activity levels also noticeably decreased. Figure 1 shows changes in some of the professors’ habits when virtual learning was implemented.

The evaluation of professors’ QWL showed that, 2 months after remote working and social distancing, they reported being satisfied with work. Table 1 presents the QWL criteria and items evaluated in this study and the mean score attributed by professors. The most positively evaluated criteria was C3 — social interaction at work —, demonstrating satisfaction with interpersonal relationships at work.

**Table 1:** Mean scores and standard deviations (SDs) of quality of working life (QWL) criteria attributed by professors, Brasília - 2020

QWL - Criteria	Mean ± SD
C1 - Working conditions	
1. How satisfied are you with your weekly workload (number of hours worked)?	3.5 ± 0.96
2. How do you feel about your workload (amount of work)?	3.33 ± 1.05
3. How do you feel about the use of technology at work after the suspension of in-person classes?	3.83 ± 1.01
4. How do you feel about the tiredness caused by your job?	2.78 ± 0.86
C2 - Use of capacities at work	
5. Are you satisfied with your autonomy (opportunity to make decisions) at work?	3.88 ± 0.97
6. Are you satisfied with the importance of the task/work/activity you perform?	4.05 ± 0.99
7. How do you feel about your polyvalence (possibility of performing various tasks and activities) at work?	3.8 ± 0.94
8. How satisfied are you with your performance review according to the students’ semiannual evaluation?	3.93 ± 0.89
9. How do you feel about the responsibility placed on you?	4.1 ± 0.78
C3 - Social interaction at work	
10. How do you feel about the existence of discrimination (whether it be social, racial, religious, sexual, or other) at your workplace?	4.03 ± 1.03
11. How do you feel about your relationship with colleagues and superiors at your workplace?	4.35 ± 0.74
12. How do you feel about the commitment of your team and colleagues to work?	4.2 ± 0.76
13. How satisfied are you with the valorization of your ideas and initiatives at work?	4.1 ± 0.74
C4 - Space work occupies in the professor’s life	
14. How satisfied are you with the influence of work on your family life/routine?	3.43 ± 0.96
15. How satisfied are you with the influence of work on your leisure possibilities?	3.33 ± 1.02
16. How satisfied are you with your work and rest periods?	3.2 ± 1.04

When correlating changes in behavioral patterns with each evaluated criterium, we observed that patterns of physical activity and fruit and vegetable intake were associated with higher or lower job satisfaction (Table 2). Professors who increased or decreased their weekly physical activity levels presented higher satisfaction with the use of capacities at work and social interaction criteria when compared to those who maintained their routines.

**Table 2:** Relationship of behavioral patterns of physical activity, fruit intake, food delivery orders, and hours of sleep with the 4 criteria of quality of life evaluated during the second month of the COVID-19 pandemic

	QWL score			
	C1	C2	C3	C4
Physical activity				
Maintained	3.25 ± 1.06	3.9 ± 0.8*	3.75 ± 0.75*,^†^	3.17 ± 1.75
Decreased	3.5 ± 1	4.4 ± 0.8^†^,^‡^	4.75 ± 1^†^,^‡^	3.33 ± 1
Increased	3.25 ± 1	3.8 ± 0.6*	4.5 ± 0.5^‡^	3.67 ± 1.33
Fruit intake				
Maintained	3.5 ± 0.75*	4.2 ± 0.6	4 ± 1.25	3.67 ± 1^†^
Decreased	2.75 ± 1.25^‡^	3.4 ± 1.3	4.5 ± 0.88	3 ± 1.5
Increased	3 ± 0	3.6 ± 0	3.5 ± 0	2.33 ± 0^‡^
Food delivery orders				
Maintained	3.5 ± 0.88	4.2 ± 0.75	4 ± 1.44	3.33 ± 1
Decreased	3.5 ± 0	4 ± 0	4 ± 0	4 ± 0
Increased	3 ± 1	4 ± 1.4	4.25 ± 0.88	3 ± 1.83
Hours of sleep				
Maintained	3.5 ± 0.88	4.2 ± 0.75	4 ± 1.44	3.33 ± 1
Decreased	3.5 ± 0	4 ± 0	4 ± 0	4 ± 0
Increased	3 ± 1	4 ± 1.4	4.25 ± 0.88	3 ± 1.83

Data presented as medians and interquartile ranges.

C1 = working conditions; C2 = use of capacities at work;

C3 = social interaction at work; C4 = space work occupies in the professor’s life; QWL = quality of working life.

* Significant difference when compared to a behavior that decreased, in the evaluated criteria, for significant differences p ≤ 0.05.

† Significant difference when compared to a behavior that increased, in the evaluated criteria, for significant differences p ≤ 0.05.

‡ Significant difference when compared to a behavior that was maintained, in the evaluated criteria, for significant differences p ≤ 0.05.

Professors who maintained their fruit and vegetable intake during the 2 months of social distancing presented higher satisfaction with working conditions than those who decreased this intake. The criterium regarding the space work occupied in the professor’s life was also related to fruit and vegetable intake. Those who maintained their eating habits regarding this aspect reported being more satisfied than those who increased fruit and vegetable intake. Changes regarding the consumption of prepared meals and hours of sleep did not have significant impacts on the professors’ job satisfaction.

## Discussion

Social distancing led to changes in the work process and lifestyle habits of faculty members. Important habits for the maintenance of QWL, in particular, suffered harmful changes, such as decreases in the number of hours of sleep, fruit and vegetable intake, and frequency of physical activity, as well as increases in food delivery orders. Other Brazilian and international studies evaluated lifestyle changes after 1 to 2 months of lockdown, similarly to this study.^12-15^

A decrease in the amount of sleep during the pandemic was reported by other studies such as that by Barros et al.,^13^ performed in Brazil, and by Wang et al.,^16^ performed in China. In these studies, part of the population reported that during lockdown, sleep problems appeared or worsened. Lack of sleep or poor sleep quality affect the mood and increase anxiety and depression. This could affect work quality and performance. Physical inactivity or sedentary behavior are known to lead to reductions in sleep duration. Therefore, the reduction in physical activity caused by lockdown may be one of the causes of reductions in sleep duration.^17^

The excessive use of screens may also disturb sleep. Professors had a large increase in the number of hours they spent at the computer or using other electronic devices. The increase in total screen time may have elevated stress levels, hampering the relaxation process towards rest and interfering with the amount of sleep.^18^ Continuous reductions in the number of hours of sleep may have decreased QWL and, in chronic manner, decreased job satisfaction even further.^19^

Reduced physical activity levels during the pandemic have been documented by studies such as the one by Martinez et al.,^12^ which also showed a decrease in physical activity levels in the Brazilian population during lockdown. In the study by Martinez et al.,^12^ the reduction in physical activity levels was justified by gym closures and insecurity regarding the contamination with COVID-19 during training. However, options such as home-based physical activity or activities in open spaces were rarely adopted, which was possibly due to the closure of public parks, but mainly to the fact that physical activity was associated with a specific place.

The study showed that approximately 30% of the sample reported having reduced physical activity levels in the first months of the pandemic. However, this study did not employ a specific instrument for interpreting the quantity and quality of movement,^20^ which makes the situation of faculty members even more concerning: other studies have demonstrated a lack of intensity and volume in physical activities performed by this population. This could increase the risk of psychological and metabolic diseases.^21^

The change in the way of working (from in-person to remote) may have also contributed to this reduction, since working from home may lead to poor time management and difficulty establishing work-life boundaries; in addition to household chores, these aspects overload professors and do not allow time for other activities.^22^ Regular physical activity provides various benefits to daily life, such as higher energy levels, lower stress levels, improved sleep, higher self-esteem, as well as reductions in muscle pain and anxiety.^23^

Other reasons for a reduced physical activity level among faculty members may have been the time required for adapting lectures and responsibilities with children and the household. Socialization itself, which could previously take place during physical activity, became virtual during the pandemic and did not require commuting or physical demands. Even if virtual socialization may have fulfilled some social interaction needs, the reduction in physical activity levels was associated with increases in anxiety and depression.^12^ Nevertheless, we believe that this effect may be transitory, and continuous low levels of physical activity may lead to increased stress, self-dissatisfaction, and decreases in QWL.^20^

Regarding the participants’ satisfaction with social interactions, we observed that professors who increased or decreased physical activity levels during the pandemic were more satisfied with social interactions than those who maintained the same level of physical activity. Once more, we see the effect of adapting to changes in the professors’ routines and work processes. For some professors, decreasing physical activity levels provided higher social interaction; for others, increasing these levels affected their satisfaction with social interaction during the pandemic. This is closely related with the kind of physical activity performed by participants; it could be performed through indoors isolated or group video classes and/or outdoors.^24,25^ As studies indicate, the lack of physical activity in the long-term decreases job satisfaction and may affect interpersonal relationships.

A decrease in fruit and vegetable intake may be associated with changes in the food environment. The food environment consists in food retailers, restaurants, cafeterias, or fast-food chains professors use as sources of food or meals. The closure of restaurants and street fairs, among others, may hinder access to healthy foods.^26^

A study performed in Poland reported the fear of the population, particularly women, of shopping at supermarkets during the pandemic.^14^ Therefore, the increase in delivery orders may be related to an insecurity in leaving the house and contracting COVID-19, as well as to restrictions to non-essential services enforced during lockdown. The eating habits of professors prior to the pandemic allowed wide access to different foods. Therefore, the responsibility of preparing meals and cooking daily may have generated, as an alternative, the consumption of ready-made meals. Particularly in Brazil, this was made possible by food delivery apps. In other countries such as Italy, food delivery orders decreased, since their delivery system is not equivalent to that in Brazil.^15^ The Brazilian Ministry of Health^18^ suggests that individuals eat fruits and vegetables as part of their meals on a daily basis, in addition to fruits in desserts or snacks. Therefore, balanced nutrition, together with regular physical activity, provide various benefits that result in better health and consequently increase quality of life.^19^

When analyzing the impact of changes in professors’ eating habits, we noticed that those who maintained their fruit and vegetable intake were more satisfied with working conditions when compared to those who decreased this intake, highlighting that good eating habits positively influence QWL.^19^ On the other hand, those who increased fruit intake had lower levels of satisfaction with the space work occupied in their lives. Maybe the pandemic shifted the professors’ perspective on the importance of work versus health, thus increasing the value of a good diet as a health care measure and, in a way, devaluing work in their life perspective. We observed that remote working changed (increased or decreased) the intake of certain foods. This was also observed by another study, which showed a correlation between changes in the work process and changes in appetite.^15^ Lockdown may have also led to changes in eating habits. Ammar et al.^27^ reported that most individuals studied during the pandemic increased their calory intake, whereas Sidor & Rzymski^14^ reported that eating habits and food intake were correlated with body mass index (BMI). Overweight or obese individuals presented an increasing trend of food intake; those with normal or low BMI tended to decrease food intake even further.^14^

In general, faculty members reported being satisfied with various aspects of work. Despite the changes and challenges, participants were able to maintain an optimistic outlook on work. As data referring to QWL were collected in the first phase of lockdown, we cannot guarantee that this satisfaction was maintained in the following months. Prolonged lockdown itself may have caused various changes, such as increases in anxiety and depression. The use of electronic devices for many months and their impact on professors’ lives should still be studied.^28,29^

Possible limitations of this study are related to memory bias, since participants had to remember their lifestyle habits before social distancing, and selection bias, because this is a convenience sample and non-respondent professors could have different opinions from the respondents. We suggest that longitudinal studies be conducted for assessing faculty members’ satisfaction and lifestyle habits in more detail throughout the duration of remote working, in order to verify the progress of these changes during lockdown. The information contained in this study is important for understanding the impact of lockdown and remote working on faculty members. This research also evaluated different aspects of QWL, comparing the influence of lifestyle changes on QWL. The COVID-19 pandemic transformed the way we teach, learn, and live.

## Conclusions

During social distancing, faculty members were satisfied with work, specifically regarding criteria of working conditions, use of capacities at work, social interaction at work, and the space work occupied in their lives. The level of work satisfaction measures QWL. Changes in lifestyle habits during the pandemic were shown to influence work satisfaction. The decrease in fruit and vegetable intake promoted a reduction in satisfaction with working conditions. On the other hand, decreasing physical activity levels promoted higher satisfaction with the criteria considering the use of capacities at work and social interaction at work. Increasing physical activity levels during the pandemic also had a positive effect on the criterium of social interaction at work.

Regarding lifestyle habits, we noticed that during lockdown, professors decreased the amount of sleep, increased the consumption of home-delivered meals, decreased fruit and vegetable intake, and decreased weekly physical activity levels. These changes are related to the adaptation to lockdown, thus the maintenance of remote working was able to modify the daily routines reported by participants. We highlight that changes related to habits, despite not showing a large impact on job satisfaction, are known to decrease QWL, and may harm the professors’ health in the long-term and decrease job satisfaction.
